# Supplementary material for: A maize mutant in the glutamate receptor-like dwarf13 is modified by cis-acting natural variation and a cornichon homolog
Source: PLoS Genet. 2025 Dec 31;21(12):e1012006. doi: 10.1371/journal.pgen.1012006 (PMC12782448; doi:10.1371/journal.pgen.1012006)
Supplement: Attachment — Submitted filename: Response to reviews PG Oct30.docx [file pgen.1012006.s020.docx]

**Response to reviews**

All responses are provided in line, italicized, and in a distinct blue color to make them maximally easy to distinguish from the editor’s and reviewer’s comments.

PGENETICS-D-25-00922
The semi-dominant D13-1 mutant of maize is modified by strong cis-acting natural variation in transcript abundance.
PLOS Genetics

Dear Dr. Dilkes,

Thank you for submitting your manuscript to PLOS Genetics. After careful consideration, we feel that it has merit but does not fully meet PLOS Genetics's publication criteria as it currently stands. Therefore, we invite you to submit a revised version of the manuscript that addresses the points raised during the review process.

Please submit your revised manuscript within 60 days Dec 27 2025 11:59PM. If you will need more time than this to complete your revisions, please reply to this message or contact the journal office at [plosgenetics@plos.org](mailto:plosgenetics@plos.org). When you're ready to submit your revision, log on to <https://www.editorialmanager.com/pgenetics/> and select the 'Submissions Needing Revision' folder to locate your manuscript file.

Please include the following items when submitting your revised manuscript:

* A rebuttal letter that responds to each point raised by the editor and reviewer(s). You should upload this letter as a separate file labeled 'Response to Reviewers'. This file does not need to include responses to any formatting updates and technical items listed in the 'Journal Requirements' section below.
* A marked-up copy of your manuscript that highlights changes made to the original version. You should upload this as a separate file labeled 'Revised Manuscript with Track Changes'.
* An unmarked version of your revised paper without tracked changes. You should upload this as a separate file labeled 'Manuscript'.

If you would like to make changes to your financial disclosure, competing interests statement, or data availability statement, please make these updates within the submission form at the time of resubmission. Guidelines for resubmitting your figure files are available below the reviewer comments at the end of this letter.

We look forward to receiving your revised manuscript.

Kind regards,

Tatiana Giraud
Academic Editor
PLOS Genetics

Quan Wang
Section Editor
PLOS Genetics

Aimée Dudley
Editor-in-Chief
PLOS Genetics

Anne Goriely
Editor-in-Chief
PLOS Genetics

**Additional Editor Comments:**

We have now received two referee reports for your manuscript on cis-acting natural variation in transcript abundance impacting the phenotype of the semi-dominant D13-1 mutant of maize, which encodes a defective ionotropic glutamate receptor. Both referees found the manuscript valuable and original; however, concerns were raised, in particular about the clarity of the objectives and main results of the study, and the lack of broader context in the discussion.

We agree that the study would be a nice addition to PLoS Genetics but that the goals and novelty of the paper are unclear. It would be better to begin the abstract and the introduction by general statements on the scientific topics rather than on specificities of the mutants, avoiding abbreviations, especially without defining them, to provide clearer take-home messages on the results and their importance, and to render globally the abstract and title easier to understand for non-specialist readers. The introduction and discussion also could better convey the main results and their importance.

I would encourage resubmission if you are able to revise the manuscript along these lines. The referees also provide a list of excellent additional suggestions, which should also be addressed.

*We have edited the Abstract, Introduction, and Discussion to clarify the study’s purpose and value in response to this and reviewer’s suggestions. We have, where possible, sought to make the paper more obviously exciting to non-plant readers and non-specialists. We edited the introduction for brevity to focus the reader on details needed to interpret the results and not “split” or “dilute” focus as requested by the reviewers. We changed the title of the manuscript in response to this and the reviewer’s suggestion.*


**Journal Requirements:**

If the reviewer comments include a recommendation to cite specific previously published works, please review and evaluate these publications to determine whether they are relevant and should be cited. There is no requirement to cite these works unless the editor has indicated otherwise.

*The suggested references are excellent and we have included them in the revised manuscript.*

1) Please ensure that the CRediT author contributions listed for every co-author are completed accurately and in full.
At this stage, the following Authors/Authors require contributions: Amanpreet Kaur, Rajdeep Singh Khangura, and Brian P Dilkes. Please ensure that the full contributions of each author are acknowledged in the "Add/Edit/Remove Authors" section of our submission form.
The list of CRediT author contributions may be found here: <https://journals.plos.org/plosgenetics/s/authorship#loc-author-contributions>

*We have added contributions*

2) Please provide an Author Summary. This should appear in your manuscript between the Abstract (if applicable) and the Introduction, and should be 150-200 words long. The aim should be to make your findings accessible to a wide audience that includes both scientists and non-scientists. Sample summaries can be found on our website under Submission Guidelines:
<https://journals.plos.org/plosgenetics/s/submission-guidelines#loc-parts-of-a-submission>

*done*

3) Please upload all main figures as separate Figure files in .tif or .eps format. For more information about how to convert and format your figure files please see our guidelines: 
<https://journals.plos.org/plosgenetics/s/figures>

*done*

4) We are unable to open the following Supporting Information file: Supplemental Figures 1-5.zip. Please kindly revise as necessary and re-upload.

*New versions of these files were uploaded*

5) Some material included in your submission may be copyrighted. According to PLOSu2019s copyright policy, authors who use figures or other material (e.g., graphics, clipart, maps) from another author or copyright holder must demonstrate or obtain permission to publish this material under the Creative Commons Attribution 4.0 International (CC BY 4.0) License used by PLOS journals. Please closely review the details of PLOSu2019s copyright requirements here: PLOS Licenses and Copyright. If you need to request permissions from a copyright holder, you may use PLOS's Copyright Content Permission form.
Please respond directly to this email and provide any known details concerning your material's license terms and permissions required for reuse, even if you have not yet obtained copyright permissions or are unsure of your material's copyright compatibility. Once you have responded and addressed all other outstanding technical requirements, you may resubmit your manuscript within Editorial Manager. 
Potential Copyright Issues:
i) Please confirm (a) that you are the photographer of 2A, or (b) provide written permission from the photographer to publish the photo(s) under our CC BY 4.0 license.

*This was taken by the primary author*

ii) Figure 1B. Please confirm whether you drew the images / clip-art within the figure panels by hand. If you did not draw the images, please provide (a) a link to the source of the images or icons and their license / terms of use; or (b) written permission from the copyright holder to publish the images or icons under our CC BY 4.0 license. Alternatively, you may replace the images with open source alternatives. See these open source resources you may use to replace images / clip-art:
- <https://commons.wikimedia.org>
- <https://openclipart.org/>.

*This was drawn by the primary author and so no clip art attribution is necessary.*

6) Please amend your detailed Financial Disclosure statement. This is published with the article. It must therefore be completed in full sentences and contain the exact wording you wish to be published.
1) State the initials, alongside each funding source, of each author to receive each grant. For example: "This work was supported by the National Institutes of Health (####### to AM; ###### to CJ) and the National Science Foundation (###### to AM)."

2) State what role the funders took in the study. If the funders had no role in your study, please state: "The funders had no role in study design, data collection and analysis, decision to publish, or preparation of the manuscript."

3) If any authors received a salary from any of your funders, please state which authors and which funders..
If you did not receive any funding for this study, please simply state: u201cThe authors received no specific funding for this work.u201d

7)  Please send a completed 'Competing Interests' statement, including any COIs declared by your co-authors. If you have no competing interests to declare, please state "The authors have declared that no competing interests exist". Otherwise please declare all competing interests beginning with the statement "I have read the journal's policy and the authors of this manuscript have the following competing interests"

*No competing interests.*

**Reviewers' comments:**

Reviewer's Responses to Questions

**Comments to the Authors:
Please note here if the review is uploaded as an attachment.**

Reviewer #1: The paper entitled “The semi-dominant D13-1 mutant of maize is modified by strong cis-acting natural variation in transcript abundance”, provide evidence strengthening the link between glutamate receptor-like channels pathway and growth regulation in plants. And by using a population genetics approach, the authors deepen our understanding on how genetic variation can regulate a complex trait.

In one hand, the paper yield information about GLR11/D13-1 allele regulation in maize. Such as the modulation of GLR11 transcript abundance due to particular SNPs located in cis. Or by the very plausible interaction with a CORNICHON stabilizing protein (encoded by the Zm00001d036084 gene), which constitutes proof of GLRs and CNIH interaction for the first time in maize (at least at gene level). Using as a model the GLRs pathway, in the other hand, some of the effects of the genetic variation over a polygenic and complex trait are revealed.

However, the main objective of the authors is unclear. The title, the abstract and the paper organization, seem to me inclined to the second (using the GLRs pathway as a model to study the influence of natural variation in a selected trait). But the introduction and the discussion fail delimiting and organizing both subjects so that the reader can follow the main theme. Alternatively, if the aim is focused on the GLR11 gene characterization, the introduction must deepen in GLRs prior characterization and functions (specially their involvement with distinct hormones, since the link with growth may reside there).

*The paper uses natural variation in combination with induced mutants to identify new details about the role of GLRs in plant growth and the effects of genetic variation on polygenic traits. We reorganized the abstract and introduction to move from general to specific to make this easier. We added more details about GLRs in the introduction to tilt the focus of the reader towards these genes and their roles in plants and other organisms. This included the addition of multiple primary research papers (as suggested below). We edited the introduction for brevity so that the focus on GLR was more clear.*

Although some of the results reported on the paper have already been observed in other plant species as Arabidopsis, Physcomitrium and rice (as the interaction of CNIH or their role in balancing defense and growth), it does contain novel data for Zea mays. Which contributes to the characterization and classification of this important receptors. I consider that the strongest aspects of the paper are related to GLR characterization in maize (including the cis-acting regulation revealed by natural genetic variation). And I highlight that the authors offer a list of the GLR genes in maize, a basic foundation for further studies in the area. The study of genetic variation influence on a trait, is also interesting. However, it remains uncertain whether a general model of the mechanisms linking genetic variation and gene activity can be established based on these findings. Since it appears to be highly diverse and depending on the characteristics of the specific gene.

*Just as a note for this reviewer regarding the possibility of a defense-growth balance mediated by the d13 gene: We found no evidence of the D13-1 allele balancing defense and growth. Though not presented in this manuscript, we compared the DEGs detected in the thesis of the primary author (Kaur 2020) to gene expression from maize mutants with hyperactive defense responses and saw no overlap greater than expected by random chance. We suspect, strongly, that as more mutants in GLRs are described it will be revealed that GLR in plants operate in and through a variety of pathways and that GLR functions will not always overlap. The repeated discovery of mechanical wounding and insect defense responses across species may owe to a fortuitous overlap in function for those specific GLRs. Work to comprehensively test all GLRs will determine if d13 is truly unusual or if GLRs are involved in multiple, independent, signal transduction networks in plants. In response to this reviewer, we included discussion of the GLR roles in defense signaling (as suggested below) and added this latter hypothesis to the discussion.*


Major issues.
1. The core theme of the manuscript needs to be more clearly articulated. Both the Introduction and Discussion sections should be revised to consistently align with and reinforce this central theme.

*We revised both sections to reinforce the insights of the paper for growth control in plants, GLR function, and placed the study in a complex-trait context last.*

2. The manuscript would benefit significantly from a clearer delineation of its overall direction and the specific subjects explored. While the interconnected nature of these aspects is important, clearly setting these boundaries will make the paper more accessible to readers and effectively highlight the impact of the claims presented.

*As suggested below, reordering sections improved the flow of the discussion. In response to this, comments below, and the comments above we focused the introduction. Thank you for the suggestion.*

3. Despite aiming to establish the influence of genetic variation on a selected trait as a main topic, the introduction and neglect to establish the frame to contextualize the subject significance. Although the it does talk briefly about the link between genetic variation and biological pathway regulation, several other prior topics as GLR functions or plant growth regulation dilutes the attention from this central theme. I suggest to always place first the central theme, and once is fully presented or discussed, particularities of the secondary subject can be addressed in a deep but concise way.

*We reordered the introduction to focus the reader on the D13 mutant and GLR function first and then introduce the FOAM approach and study of natural variation second. This puts growth, mutant, GLR as primary and method/complex traits as secondary in the introduction. Thank you for the suggestion.*

4. The discussion fails to offer broad conclusions on mechanisms of how variation in DNA sequences can influence a trait. Instead it alternates the focus on the particularities of GLR regulation (such as the formation of tetramers or the known characteristics and functions of CORNICHON genes). To avoid diluting the relevance of how genetic variation influencing traits, GLR specific results can be used as examples to make general points.

Alternatively, the main focus of the paper can be GLR11 gene regulation and function in maize. While the linkage of natural genetic variation and trait regulation can come as secondary theme. In this case, GLR11 phenotype can be broadly discussed in the context of known GLR functions.

*We reordered the discussion as suggested. We sharpened the discussion of known GLR functions and reordered the general (above) and specific (below) insights. We focused first on the effects and mechanisms at play in D13-1, placing the approaches used and this study in a broader complex-trait genetics frame last, as suggested. This was achieved both by reordering paragraphs within sections and reordering sections so that the discussion of complex genetics analysis no longer broke up the discussion of D13 allele mechanisms. This allowed us to delete some statements that were repeated and tighten up the discussion. Thank you for the suggestion.*

5. Following this idea, the paper could benefit from a deeper integration with evidence of GLR activities in other species. Although the paper from Yi, et al. (2022) is mentioned, there are other papers linking GLRs with defense and (directly or indirectly) with growth. For example, the papers showing that the systemic signaling against herbivory is mediated by the glutamate receptor-like channels (Mousavi, et al. 2013), demonstrate the link with the Jasmonate pathway. Strong pieces of evidence accumulated over the years (such as growth defects in jasmonate signaling mutants), have sustained the defense-growth trade-off. So that the involvement of GLRs upstream jasmonate signaling and in defense in general (Li, et al. 2013 & Toyota, et al. 2018), indirectly link them with growth. The work from Hernández-Coronado, et al (2022) represents another piece of evidence linking the GLRs and growth. By showing that regeneration is mediated by GLRs through the SA pathway, the relationship of GLRS with distinct hormone pathways beyond Brassinosteroids may account for the growth defect phenotype of the D13-1 mutant.

*We incorporated these references, and the demonstration of GLR roles in defense signaling in rice, Arabidopsis and others into the introduction and expanded on this idea in the discussion.*

*Worth noting here (said in different words above) that there is no evidence in either metabolite profiling or gene expression analyses for an upregulation of defense responses in D13-1 mutants (Kaur, 2020). We decided not to emphasize this too strongly since D13-1 is a dominant allele and is currently the odd GLR out in the plant literature. While multiple other GLRs are clearly involved in defense (particularly chewing/mechanical damage) this one (at least this allele of this one) appears to be affecting a distinct signaling pathway. The isolation of more mutants of maize, and molecular study of multiple GLRs will either determine D13-1 to be an oddball mutation, or demonstrate that the plant GLRs are part of multiple signaling pathways and function independently.*

Recommended references.
Yu, B., Wu, Q., Li, X., Zeng, R., Min, Q. and Huang, J. (2022), GLUTAMATE RECEPTOR-like gene OsGLR3.4 is required for plant growth and systemic wound signaling in rice (Oryza sativa). New Phytol, 233: 1238-1256.

Mousavi SA, Chauvin A, Pascaud F, Kellenberger S, Farmer EE. GLUTAMATE RECEPTOR-LIKE genes mediate leaf-to-leaf wound signalling. Nature. 2013 Aug 22;500(7463):422-6. doi: 10.1038/nature12478. PMID: 23969459.

Li F, Wang J, Ma C, Zhao Y, Wang Y, Hasi A, Qi Z. Glutamate receptor-like channel3.3 is involved in mediating glutathione-triggered cytosolic calcium transients, transcriptional changes, and innate immunity responses in Arabidopsis. Plant Physiol. 2013 Jul;162(3):1497-509. doi: 10.1104/pp.113.217208.

Hernández-Coronado M, Dias Araujo PC, Ip PL, Nunes CO, Rahni R, Wudick MM, Lizzio MA, Feijó JA, Birnbaum KD. Plant glutamate receptors mediate a bet-hedging strategy between regeneration and defense. Dev Cell. 2022 Feb 28;57(4):451-465.e6. doi: 10.1016/j.devcel.2022.01.013.

6. The name of both the GLR gene and the CORNICHON gene, bust be maintained as the names previously attributed in the literature. So that the D13-1 mutant should be named as ZmGLR11-1. And the TOD1 gene as ZmCNIH. I do understand that those mutants were found by forward genetics and named according to the experiment. But once the identity if corroborated, names can be changed for clarity and uniformity in nomenclature, as well as to give credit to previous research.

*There is no pre-existing nomenclature for either GLRs or CORNICHON homologs in maize. The name ZmGLR11 is the name given to this family member by the primary author in her thesis. These genes have not been comprehensively named by others. She found the molecular identity of the D13 gene by forward genetics and then, as the reviewer points out, needed a systematic naming of all the GLRs to make sense of them and place the d13 gene in context. So, we generated a family-wise set of names here and gave d13 a GLR number which appears only in supplemental table 10 (although some in the maize community will chafe at this and would want it uniquely named as d13 only and _not_ also named GLR11). Currently, at MaizeGDB the D13 gene is not named anything other than it’s computer-generated ID. So, we are establishing the names of these genes now, here, in this manuscript. There is not a prior paper to cite for this.*

*The same is true of the two CORNICHON homologs in maize. Neither had been given a name yet. So, our forward genetic gene names are appropriate as used. We have also provided a comprehensive “family” name for each to allow people to work in that way as well.*

Finally, the impact of the paper can be increased by having the GLR name in the title.

*We modified the table as suggested. As stated above, this paper is defining the nomenclature so D13 is the GLR name. We added reference to the fact that the D13 gene is a GLR. The nomenclature is provided by this paper and there is, at the time of this writing, no pre-existing nomenclature is in the literature or at MaizeGDB.*

Minor issues:

1. Introduction for GLRs cites several reviews, but not original papers. As mentioned before, I think the paper can benefit from a deeper discussion of GLR functions. For which original research papers characterizing GLR functions in plants may come useful.

*We reworked the introduction and incorporated suggested references from both reviewers as well as additional primary references about work on GLR as suggested elsewhere in this reviewer’s comments.*

2. The introduction could be more concise in its presentation of growth as a complex trait. While this point is important, the explanation is longer than necessary and could be tightened to improve flow and reader engagement.

*We edited and reordered this section for clarity, in line with this and the other reviewer’s suggestions.*

3. The introduction can be improved by addressing further the role of genetic variation in gene expression and regulation by relevant studies in yeast and humans.

*The introduction was edited and reordered and focused more directly on d13 as requested by this reviewer.*

4. Page 9, last sentence. Zm00001d03608 is missing a number.

“Transcript abundance data for the Zm00001d03608 gene in the inbreds constituting the maize association panel were obtained from four above-ground tissues [germinating shoot…”

*Thank you for catching this, the wayward “4” has been returned to Zm00001d036084.*

Reviewer #2: In this paper, the authors are investigating the molecular bases of the semi-dominant dwarf maize mutant D13-1, caused by a SNP in the coding sequence that mutate an otherwise conserved alanine into a guanine in the hinge that opens and closes the gate of a glutamate receptor-like ion channel (GLR). As this phenotype is semi-dominant and can be modified or even completely reversed in some genetic backgrounds, it is most likely a complex phenotype partially determined by some complex regulatory interactions. The authors thus used a F1 association mapping approach by crossing a D13-1 mutant with hundreds of maize lines to investigate the molecular bases of dwarfism associated with D13-1. They performed association studies with dwarfism and gene expression levels to uncover modifier loci, i.e. the mutations playing a regulatory role and affecting the dwarf phenotype. They identified one trans-modifier loci, tod1, and several independent cis-regulatory loci on the d13 locus that affect the mutant severity. The study is sound and well-presented, I only have minor comments.

*Thank you!*

Comments
1. Please reference the subpanel of the figure when citing it in the text, it will make it easier for the reader to navigate the paper (especially for figures 4-6 and supplementary figure S1, where there are a lot of similar panels). Please also elaborate on the content of the different panels (in particular what the trats are) in Supp. Fig.1.

*All text was modified to mention the subpanel of the figures. We elaborated the traits in the legend for Supp Fig S1.*

2. For the single locus association study at d13: why did you choose a 250kb window and not a larger one, as cis-regulatory relationships can span up to 1Mb in maize?

*Linkage disequilibrium in maize generally decays much faster than 250kb (see estimates of species-wide ~10 kb on average and 10s of kb for broad subpopulations in Flint-Garcia et al., 2003; Lu et al., 2011; Pace et al., 2015). It is true that maize encodes some very storied long-distance cis effects, but these appear to be unusual cases. Even in some of the most often cited long distance enhancer examples in maize such as natural variation in long-distance cis regulatory elements at RAP2.7 affecting flowering time (vgt1), the Tb1 cis acting variation, and the B enhancer involved in B’ paramutation even those are “only” ~70kb, ~70kb, and ~100kb from their respective transcripts. So, our window is quite large (as intended) and would capture those as “cis” by our definition.*

*Like this reviewer we are thinking about this quite a bit and share the following summary of our observations to date. For all the genes that we have focused on in our work to date (published and unpublished) the highest test statistics are within this 250kb window. In our maize work we have yet to observe a case where the strongest association (for a gene of interest; dozens of genes) was outside of 50kb. This seems to indicate that vgt1, tb1, and B’ are, indeed, unusual. What we do see, often, are SNPs that pass a more permissive thresholds (e.g. our preliminary cutoff of 10-4) at greater distances than our window. We have not investigated this comprehensively, but in those cases where we have interrogated carefully, this owes to a very-large-effect cis-acting polymorphism near the transcript. So, at least in the cases we have interrogated, the physically distant SNPs do not look like cis-acting expression variation caused by variation at great distances but rather look like residual LD with very large effect alleles. Dilkes strongly suspects that strong associations detected at distances greater than 250kb will turn out to be due to inversions and other structural variation that have suppressed recombination resulting in long but local LD-suppression within a haplotype rather than causative SNP-level allele variation acting in cis at great distances (e.g. the 1Mb cited above). When we do observe strong expression level polymorphisms encoded at distances greater than 250kb they are very distant or on other chromosomes (9/10 on other chromosomes expected with 10 chromosomes of maize) and are clearly trans-acting variants. We see nothing to indicate there is pervasive long-distance cis-regulatory variants responsible for natural variation in gene expression for any of the pathways we have worked on (porphyrin, GA, BR, and single gene examples like the one presented here).*

3. For the eQTL study at d13 locus: why announcing an eGWAS approach and then referencing eQTLs in the text. You can directly say that you used an eQTL discovery approach, that would be less confusing for the reader.

*All the natural variation studies in the paper are done by GWAS including the transcript accumulation association mapping. We used the abbreviation “eQTL” a single time at this location and the abbreviation “QTL” in two other locations. In all three locations we are referring to quantitative trait loci, as they are quantitative trait loci, but none are derived from line crosses. We have un-abbreviated these turns of phrase, since there are so few of them, so as to not cause confusion and prioritized the abbreviation GWAS to focus on the association mapping approach that was used in all cases.*

4. p.9 last paragraph, “Transcript abundance data for the Zm00001d03608 gene in the inbreds”: there is a “4” missing at the end of the gene name.

*Thank you for catching this, the wayward “4” has been returned to Zm00001d036084.*

5. In Figure 4, the E. is missing in the legend.

*Thank you for catching this, the wayward “E” has been returned to the legend of Figure4*

6. Please put all occurrence of cis and trans in italic.

*Modified as suggested! Thank you for making these stand out more!*

7. Please add a data availability section with links or references to all genomic and raw expression data if they are stored in public databases. If pertinent, please mention the date at which the data were dowloaded and genome version. For the scripts, please mention the version of the packages used. Making the script publicly available would enhance the repoducibility of the paper.

*All phenotypic data are new and public and included in this manuscript. Genomic and expression data are from prior work; our reanalysis utilizes the tables published in prior work and so there is no raw data of that type newly disclosed in this paper.*

**Have all data underlying the figures and results presented in the manuscript been provided?**
Large-scale datasets should be made available via a public repository as described in the *PLOS Genetics* [data availability policy](https://nam04.safelinks.protection.outlook.com/?url=http%3A%2F%2Ftrack.editorialmanager.com%2FCL0%2Fhttp%3A%252F%252Fjournals.plos.org%252Fplosgenetics%252Fs%252Fdata-availability%2F1%2F010f019a2b876613-d2d96b30-1736-4124-af9d-e2c96c1d8ee6-000000%2FIA_FrrEgFf-zdGvtYEDmlSU9apjRz-RmA0mSG9U6j5s%3D235&data=05%7C02%7Cbdilkes%40purdue.edu%7Ca857b0a3955e4223a88a08de163a5127%7C4130bd397c53419cb1e58758d6d63f21%7C1%7C0%7C638972636923564402%7CUnknown%7CTWFpbGZsb3d8eyJFbXB0eU1hcGkiOnRydWUsIlYiOiIwLjAuMDAwMCIsIlAiOiJXaW4zMiIsIkFOIjoiTWFpbCIsIldUIjoyfQ%3D%3D%7C0%7C%7C%7C&sdata=f07j3E9RDgQ14khZHZ0A0PHPovJp5Deu3oyTyNN7j1o%3D&reserved=0), and numerical data that underlies graphs or summary statistics should be provided in spreadsheet form as supporting information.

Reviewer #1: Yes

Reviewer #2: **No:** I think all the data used are publicly available, but there are no "Data availability" section that allows to easily find them.

*All of the data are present in the supplemental files of the submitted manuscript.*

PLOS authors have the option to publish the peer review history of their article ([what does this mean?](https://nam04.safelinks.protection.outlook.com/?url=https%3A%2F%2Ftrack.editorialmanager.com%2FCL0%2Fhttps%3A%252F%252Fjournals.plos.org%252Fplosgenetics%252Fs%252Feditorial-and-peer-review-process%2523loc-peer-review-history%2F1%2F010f019a2b876613-d2d96b30-1736-4124-af9d-e2c96c1d8ee6-000000%2FQF0KewYOkHaC9Rq_gfDs2QNIx589Xaupsa8MhVXROOw%3D235&data=05%7C02%7Cbdilkes%40purdue.edu%7Ca857b0a3955e4223a88a08de163a5127%7C4130bd397c53419cb1e58758d6d63f21%7C1%7C0%7C638972636923626489%7CUnknown%7CTWFpbGZsb3d8eyJFbXB0eU1hcGkiOnRydWUsIlYiOiIwLjAuMDAwMCIsIlAiOiJXaW4zMiIsIkFOIjoiTWFpbCIsIldUIjoyfQ%3D%3D%7C0%7C%7C%7C&sdata=iV7dQq0F5WioG0Qx1qf3%2BBwx1OpnSOhMgZxsDH%2FRZhM%3D&reserved=0)). If published, this will include your full peer review and any attached files.


If you choose “no”, your identity will remain anonymous but your review may still be made public.


**Do you want your identity to be public for this peer review?** For information about this choice, including consent withdrawal, please see our [Privacy Policy](https://nam04.safelinks.protection.outlook.com/?url=https%3A%2F%2Ftrack.editorialmanager.com%2FCL0%2Fhttps%3A%252F%252Fwww.plos.org%252Fprivacy-policy%2F1%2F010f019a2b876613-d2d96b30-1736-4124-af9d-e2c96c1d8ee6-000000%2FSLf4lqzH_2jfKCzR5DKnBNV822ryZ5foySFzE1kx-EI%3D235&data=05%7C02%7Cbdilkes%40purdue.edu%7Ca857b0a3955e4223a88a08de163a5127%7C4130bd397c53419cb1e58758d6d63f21%7C1%7C0%7C638972636923643257%7CUnknown%7CTWFpbGZsb3d8eyJFbXB0eU1hcGkiOnRydWUsIlYiOiIwLjAuMDAwMCIsIlAiOiJXaW4zMiIsIkFOIjoiTWFpbCIsIldUIjoyfQ%3D%3D%7C0%7C%7C%7C&sdata=OG2PXh%2BLvsbBTcBU8WEVxI8cZWaCPh8TFhPHHXh5%2Byo%3D&reserved=0).

Reviewer #1: No

Reviewer #2: No

**Figure resubmission:**

While revising your submission, we strongly recommend that you use PLOS’s NAAS tool (<https://ngplosjournals.pagemajik.ai/artanalysis>) to test your figure files. NAAS can convert your figure files to the TIFF file type and meet basic requirements (such as print size, resolution), or provide you with a report on issues that do not meet our requirements and that NAAS cannot fix.

After uploading your figures to PLOS’s NAAS tool - <https://ngplosjournals.pagemajik.ai/artanalysis>, NAAS will process the files provided and display the results in the "Uploaded Files" section of the page as the processing is complete. If the uploaded figures meet our requirements (or NAAS is able to fix the files to meet our requirements), the figure will be marked as "fixed" above. If NAAS is unable to fix the files, a red "failed" label will appear above. When NAAS has confirmed that the figure files meet our requirements, please download the file via the download option, and include these NAAS processed figure files when submitting your revised manuscript.

**Reproducibility:**

To enhance the reproducibility of your results, we recommend that authors of applicable studies deposit laboratory protocols in [protocols.io](http://protocols.io), where a protocol can be assigned its own identifier (DOI) such that it can be cited independently in the future. Additionally, PLOS ONE offers an option to publish peer-reviewed clinical study protocols. Read more information on sharing protocols at <https://plos.org/protocols?utm_medium=editorial-email&utm_source=authorletters&utm_campaign=protocols>
