# Supplementary material for: A maize mutant in the glutamate receptor-like dwarf13 is modified by cis-acting natural variation and a cornichon homolog
Source: PLoS Genet. 2025 Dec 31;21(12):e1012006. doi: 10.1371/journal.pgen.1012006 (PMC12782448; doi:10.1371/journal.pgen.1012006)
Supplement: Attachment — Submitted filename: Resubmission 2 Editors Notes and Responses.docx [file pgen.1012006.s021.docx]

CC: "Amanpreet Kaur" [kaur60@purdue.edu](mailto:kaur60@purdue.edu), "Rajdeep Singh Khangura" [rkhangura@wisc.edu](mailto:rkhangura@wisc.edu)

PGENETICS-D-25-00922R1
A maize mutant in the glutamate receptor-like dwarf13 is modified by cis-acting natural variation and a Cornichon homolog
PLOS Genetics

Dear Dr. Dilkes,

Thank you for submitting your manuscript to PLOS Genetics. After careful consideration, we feel that it has merit but does not fully meet PLOS Genetics's publication criteria as it currently stands. Therefore, we invite you to submit a revised version of the manuscript that addresses the points raised during the review process.

Please submit your revised manuscript within by Jan 10 2026 11:59PM. If you will need more time than this to complete your revisions, please reply to this message or contact the journal office at [plosgenetics@plos.org](mailto:plosgenetics@plos.org). When you're ready to submit your revision, log on to<https://www.editorialmanager.com/pgenetics/> and select the 'Submissions Needing Revision' folder to locate your manuscript file.

Please include the following items when submitting your revised manuscript:

* A rebuttal letter that responds to each point raised by the editor and reviewer(s). You should upload this letter as a separate file labeled 'Response to Reviewers'. This file does not need to include responses to formatting updates and technical items listed in the 'Journal Requirements' section below.
* A marked-up copy of your manuscript that highlights changes made to the original version. You should upload this as a separate file labeled 'Revised Manuscript with Track Changes'.
* An unmarked version of your revised paper without tracked changes. You should upload this as a separate file labeled 'Manuscript'.

If you would like to make changes to your financial disclosure, competing interests statement, or data availability statement, please make these updates within the submission form at the time of resubmission. Guidelines for resubmitting your figure files are available below the reviewer comments at the end of this letter.

We look forward to receiving your revised manuscript.

Kind regards,

Tatiana Giraud
Academic Editor
PLOS Genetics

Quan Wang
Section Editor
PLOS Genetics

Aimée Dudley
Editor-in-Chief
PLOS Genetics

Anne Goriely
Editor-in-Chief
PLOS Genetics

**Additional Editor Comments:**

I was pleased to see that this manuscript has been carefully revised, being much clearer, and the referees also found the revisions overall satisfactory, they only had a few minor additional suggestions left. I also have additional suggestions below.
-Lines numbers are missing, which renders cumbersome to make comments for editors and referees

*We apologize for this. We added line numbers for the resubmitted manuscript*

-The wording can be wording, some examples are given below, but the text needs to be carfully edited.
-“semi-dominant mutants”: it is not the mutant or the locus that is dominant, but an allele, right? Correct all along the manuscript

*All instances of semi-dominant mutant were reworded to a construction using semi-dominant allele throughout the manuscript.*

-Abstract: explain why this mutant is interesting, what is the phenotype earlier in the abstract

*We moved the description of the mutant and used language similar to the author summary.*

-P1: “segregated for mutant and wild-type F1 hybrids”: alleles segregate, not hybrids (same P4);

*Changed in both locations.*

“epistatically controlled” does not seem right (sounds like the opposite? The loci control the expression?);

*Phenotypic effects were only visible in the presence of the D13-1 allele, thus D13-1 is epistatic to tod1.*

“linked” is ambiguous: is it physical linkage or more generally within the pathway or similar?

*Physical linkage was the intent, but the sentence was made clearer by deletion as the next sentences detail the loci.*

“encoded by natural variation” is incorrect, a locus is not encoded and natural variation in itself does not encode anything;

*Modified.*

integration of different results not of integration.

*It was unclear to us what we should modify here. We expanded the sentence to make what was integrated more explicit.*

-P2: the severity applies to the phenotype not to the mutant; what does semidominant mean? Codominant?

*Semi-dominant and codominant are not the same. Co-dominant is when both alleles express different phenotypes simultaneously in a diploid (or similar; e.g. AB blood type or some PCR markers). Semi-dominant is when the heterozygote is visible and less severe than the homozygous mutant. This is quite common among “dominant”, visible as heterozygotes, induced mutants and is the more accurate and correct description of mutant alleles that result in an intermediate phenotype as heterozygotes. They are quite common in maize because of the mutagenesis procedures that people use (pollen mutagenesis, similar to procedures in animals e.g. ENU mutagenesis in mouse from injecting testis) results in large population of heterozygous plants that are the first population anyone screens for phenotypes (same in mouse). They can be thought of as mathematically modeled as additive variation in the population genetic sense. The fact that most induced variants are recessive, but most natural variants are additive, is one of the wedges between these two families of geneticists. We love semi-dominant expression of phenotypes because it signals to us that the phenotype is in a deformable range and is likely to get worse and better when physiological conditions vary under environmental or genetic (such as here) perturbations.*

*NIH National Library of medicine (definition is a bit messy)* [*https://www.ncbi.nlm.nih.gov/medgen/1693292*](https://www.ncbi.nlm.nih.gov/medgen/1693292)

*Merriam Webster (not geneticists, but very straightforward)* [*https://www.merriam-webster.com/dictionary/semidominant*](https://www.merriam-webster.com/dictionary/semidominant)

Unclear what you mean by “provides insights into mechanisms shaped by natural selection”? what mechanisms? (same in P4). The author summary is much clearer and better written.

*Reworded in both locations*

-P5: “the allele segregates as a single locus” sounds like a pleonasm, and an allele is not a locus.

*I am delighted to learn “pleonasm” which I am sure I do all the time! Sometimes an allele can be a single locus (a big deletion) that gets rid of multiple gene models (and so multiple loci in another sense). We made the sentence more clear and explicit by modifiying it to “The D13-1 and wild-type alleles segregated 1:1 within each isogenic F1 family”*

-P6: “genetic suppression” does not seem a correct formulation; of what?

*The phenotype was completely suppressed by genetics. Reworded to more completely describe the prior work.*

-P7: again, suppression of what? “suppressed lines” also sounds incorrect, it is not the lines that are suppressed but the expression of a gene?

*Reworded to be more complete in all locations so that suppressed refers to phenotype and a line is capable of suppressing a phenotype.*

-P10: a gene does not encode another gene

*On this page we found two uses of encode. In both cases a gene is encoding a protein (in one case a homolog of something).*

-P11: the severity does not apply to heterozygotes but to their phenotype

*Clarified.*

-Keep homogeneous tenses in sentences (for example P11 we assessed… if variation WAS linked”, but also elsewhere)

*Fixed*

-P13: recombination occurs between loci not between phenotypes (here transcript level and height)

*Fixed.*

-P17: “were likely contributed” does not seem correct syntax

*It is easier to read with the “likely” removed, and so we deleted it. Always easier to read something without a contingency attached.*

*That said, we do think “likely” is more accurate since these are Bayesian estimates for the haplotype, which are not always correct, and alleles that arise within a haplotype after introgression will be lumped in with alleles that were contributed prior to introgression.*

-Do not refer to figures o tables in the discussion, they should have been explained well enough in the result section

*We have deleted all figure and table callouts in the discussion.*

-P19: the consequence on gene regulation of the mutation not the mutant

*Fixed.*

-The discussion remains hard to read for non-specialists..; It would be good to add a first paragraph and/or a conclusion broadening the context and explaining the importance of the results for the broad audience of PloS Genetics

*The discussion was reordered at the top in response to the prior round of review. So we were somewhat reluctant to modify the beginning (possibly in opposition to prior reviewer request).*

*We added a summarizing conclusion paragraph (that could be swapped to be an over-arching first paragraph).*

**Journal Requirements:**

If the reviewer comments include a recommendation to cite specific previously published works, please review and evaluate these publications to determine whether they are relevant and should be cited. There is no requirement to cite these works unless the editor has indicated otherwise.

**Reviewers' comments:**

Reviewer's Responses to Questions

**Comments to the Authors:
Please note here if the review is uploaded as an attachment.**

Reviewer #1: In this version of the paper, now entitled "A maize mutant in the glutamate receptor-like dwarf13 is modified by cis-acting natural variation and a Cornichon homolog”, the authors succeed in delivering a clear and concise story that contributes to the advance of maize genetics. The two aspects included in the original paper are still present: the regulation of a complex developmental trait by natural genetic variation; and the characterization of members of the GLR family (particularly dwarf13 and its regulation by tod1). But in this version, the ideas are well organized, making easier to follow and to understand the paper. The introduction, for example, suffered a restructuration, making it shorter and concise. While the conclusion now highlights the relevance of the findings.

I am satisfied with the answers of the authors to my concerns. They reply to each one in a very logic and simple way, and took into consideration most comments or suggestions. I really hope that this review process has contributed to strengthen the story.

I only have a minor comment:
On page 16, second paragraph, there is an error. It says, “in in all four tissues” (in is repeated).

*Thank you for catching this, there is now only one “in” in this spot.*

Reviewer #2: The authors have answered all of my questions, and have updated the abstract, introduction and discussion in a way that makes it both more understandable and more attractive for the reader.

**Have all data underlying the figures and results presented in the manuscript been provided?**
Large-scale datasets should be made available via a public repository as described in the *PLOS Genetics* [data availability policy](http://track.editorialmanager.com/CL0/http:%2F%2Fjournals.plos.org%2Fplosgenetics%2Fs%2Fdata-availability/1/010f019b0f363b59-900b46b8-d246-4737-a528-8dd277982372-000000/dzX9vVA52ebKFaSGoL4U9bzlyipk9KWQVwfVYxJABaU=241), and numerical data that underlies graphs or summary statistics should be provided in spreadsheet form as supporting information.

Reviewer #1: Yes

Reviewer #2: Yes

PLOS authors have the option to publish the peer review history of their article ([what does this mean?](https://track.editorialmanager.com/CL0/https:%2F%2Fjournals.plos.org%2Fplosgenetics%2Fs%2Feditorial-and-peer-review-process%23loc-peer-review-history/1/010f019b0f363b59-900b46b8-d246-4737-a528-8dd277982372-000000/b0rZfc8FVZfaKAOvt2Jh_GpkVItZX_MVwHP0TQoA1Fw=241)). If published, this will include your full peer review and any attached files.


If you choose “no”, your identity will remain anonymous but your review may still be made public.


**Do you want your identity to be public for this peer review?** For information about this choice, including consent withdrawal, please see our [Privacy Policy](https://track.editorialmanager.com/CL0/https:%2F%2Fwww.plos.org%2Fprivacy-policy/1/010f019b0f363b59-900b46b8-d246-4737-a528-8dd277982372-000000/Z6GX9lck_-sSOlEtR2RSHJfQQwIu9MUGOfMUFyIgt8I=241).

Reviewer #1: No

Reviewer #2: No

**Figure resubmission:**

While revising your submission, we strongly recommend that you use PLOS’s NAAS tool (<https://ngplosjournals.pagemajik.ai/artanalysis>) to test your figure files. NAAS can convert your figure files to the TIFF file type and meet basic requirements (such as print size, resolution), or provide you with a report on issues that do not meet our requirements and that NAAS cannot fix.

After uploading your figures to PLOS’s NAAS tool - <https://ngplosjournals.pagemajik.ai/artanalysis>, NAAS will process the files provided and display the results in the "Uploaded Files" section of the page as the processing is complete. If the uploaded figures meet our requirements (or NAAS is able to fix the files to meet our requirements), the figure will be marked as "fixed" above. If NAAS is unable to fix the files, a red "failed" label will appear above. When NAAS has confirmed that the figure files meet our requirements, please download the file via the download option, and include these NAAS processed figure files when submitting your revised manuscript.

**Reproducibility:**

To enhance the reproducibility of your results, we recommend that authors deposit laboratory protocols in [protocols.io](http://protocols.io), where a protocol can be assigned its own identifier (DOI) such that it can be cited independently in the future. Additionally, PLOS ONE offers an option to publish peer-reviewed clinical study protocols. Read more information on sharing protocols at <https://plos.org/protocols?utm_medium=editorial-email&utm_source=authorletters&utm_campaign=protocols>
